# Selection in coral mitogenomes, with insights into adaptations in the deep sea

**DOI:** 10.1038/s41598-023-31243-1

**Published:** 2023-04-12

**Authors:** Nina I. Ramos, Danielle M. DeLeo, Jeremy Horowitz, Catherine S. McFadden, Andrea M. Quattrini

**Affiliations:** 1 Department of Invertebrate Zoology, National Museum of Natural History, Smithsonian Institution, Washington, DC 20560 USA; 2 Department of Biology, Harvey Mudd College, Claremont, CA 91711 USA

**Keywords:** Genome evolution, Computational biology and bioinformatics

## Abstract

Corals are a dominant benthic fauna that occur across a vast range of depths from just below the ocean’s surface to the abyssopelagic zone. However, little is known about the evolutionary mechanisms that enable them to inhabit such a wide range of environments. The mitochondrial (mt) genome, which is involved in energetic pathways, may be subject to selection pressures at greater depths to meet the metabolic demands of that environment. Here, we use a phylogenomic framework combined with codon-based models to evaluate whether mt protein-coding genes (PCGs) associated with cellular energy functions are under positive selection across depth in three groups of corals: Octocorallia, Scleractinia, and Antipatharia. The results demonstrated that mt PCGs of deep- and shallow-water species of all three groups were primarily under strong purifying selection (0.0474 < ω < 0.3123), with the exception of positive selection in *atp6* (ω = 1.3263) of deep-sea antipatharians. We also found evidence for positive selection at fifteen sites across *cox1, mtMutS,* and *nad1* in deep-sea octocorals and *nad3* of deep-sea antipatharians. These results contribute to our limited understanding of mt adaptations as a function of depth and provide insight into the molecular response of corals to the extreme deep-sea environment.

## Introduction

The deep sea (> 200 m) may impose several selective pressures that necessitate biological and physiological adaptation of resident fauna, such as deep-sea corals, to seemingly harsh conditions that include cold temperatures, high hydrostatic pressure, variable oxygen levels, and limited food^1–4^. Corals have a wide geographical and vertical distribution, and species at greater depths have been able to survive in these environments despite added selective pressures that can otherwise restrict the vertical distribution of shallow-water species^5,6^. Several coral groups have been documented to have ranges that extend from the intertidal zone to depths > 6000 m^7–9^. However, there is still relatively little known about the evolutionary processes in deep-sea corals that enable their success at greater depths^10^. Investigating underlying differences at the genomic level may elucidate insight into the molecular adaptations of corals to the extreme deep-sea environment.

Mitochondrial (mt) genomes are widely used within phylogenetic and phylogeographical studies to assess the evolutionary history of taxa^11^. Across Metazoa, mt genomes are typically composed of 13 protein-coding genes (PCGs) that encode many of the subunits responsible for generating energy through oxidative phosphorylation (OXPHOS)^12^. Genes within the mt genome have a significant impact on the metabolic pathways and functions of an organism and thus environmental pressures associated with depth have the potential to influence nucleotide substitutions and drive adaptation^4^. Recent studies of deep-sea marine invertebrates have shown evidence for sites of adaptive evolution within the mt genome. These sites of positive selection include the PCGs *atp6, cob, cox1, cox3,* and *nad1-5* in the alvinocaridid shrimp *Shinkaicaris leurokolos*—endemic to hydrothermal vent and cold seep environments^13^—and within *atp6, cob, cox2, nad2, nad4, and nad5* of mussels in the subfamily Bathymodiolinae, which inhabit chemosynthesis-based ecosystems (hydrothermal vents, cold-seeps, organic falls)^14^. While research about mt adaptations in the deep sea has been published, only one study was focused on selection in a deep-sea species within the subphylum Anthozoa—the actiniarian sea anemone *Bolocera* sp.^4^. These studies demonstrate that the mt genome is under positive selection in deep-sea species when compared to shallow-water relatives of these groups and thus support the hypothesis of adaptive mt evolution to the selective pressures of the deep-sea environment.

For most metazoans, the mt genome exhibits faster evolutionary rates than nuclear DNA (nDNA) making it useful as a genetic marker for evolutionary studies. However, anthozoans uniquely defy that standard with observed rates of mitochondrial evolution an order of magnitude slower than other invertebrates and slower than that observed within their nDNA^15,16^. Anthozoans also include a variety of unique characteristics among their mt genomes. The class Octocorallia (soft corals, sea fans, sea pens) includes an additional PCG unidentified in any other metazoan mt genome, the *mtMutS* gene^17^, which is involved in DNA mismatch repair^18^. Selection tests conducted by Muthye et al.^19^ found *mtMutS* to be one of the least conserved PCGs in the mt genome and provided evidence that its presence contributes to the low rates of mt sequence evolution in octocorals (see also Bilewitch and Degnan^18^). Furthermore, Hogan et al.^20^ described the first report of a circular bipartite mt genome in Cnidaria within an unknown species of *Umbellula* octocoral (also included in this study). While it remains unclear why or how multipartite genomes arise, their presence in other Metazoa have invoked hypotheses such as greater gene expression efficiency^21^ and specialized functions that may be selected for and advantageous in extreme environments^20,22,23^. Two orders of the class Hexacorallia included in this study, Scleractinia (stony corals) and Antipatharia (black corals), exhibit group I introns within the genes of *cox1* (e.g., homing endonuclease; present in Antipatharia) and *nad5,* further contributing to the unique set of factors to consider when assessing the adaptive evolution of coral mt genomes^24,25^.

In this study we present a phylogenomic framework to assess whether genes associated with cellular-energy functions are under positive selection across depth. We performed a series of selection tests on the mitochondrial PCGs for deep-sea and shallow-water species of Octocorallia, Scleractinia, and Antipatharia to evaluate mitogenomic differences between shallow (< 200 m) and deep-sea (> 200 m) habitats. In utilizing this approach, results from this study contribute to the limited understanding of the evolutionary response of corals to high environmental stressors in the deep sea and enable assessment of genes that may contribute to their success in that environment.

## Methods

### Data and alignments

The majority of mitochondrial genomes (n = 122) included in this study were captured as off-target reads from the target-capture sequence data in Quattrini et al.^26,27^. Methods of assembly and annotation are described in detail in Quattrini et al. (in review)^28^ and Muthye et al.^19^. Additional mt genomes for octocorals (n = 14) and scleractinians (n = 5) were downloaded from GenBank and included in the dataset. The final individual datasets for each group consisted of 84 species of Octocorallia, 44 Scleractinia, and 13 Antipatharia (Supplementary Table S1). Of those taxa, 25, 11, and six were deep-sea corals, respectively. This classification was made based on the criteria that the sampled specimen from which the mt genome was sequenced was collected below 200 m depth (see Hogan et al.^20^, Quattrini et al.^27^) and is most common at depths > 200 m according to OBIS (https://obis.org). For each taxonomic group, PCGs were aligned using MAFFT v7.481^29^ with the L-INS-I method to attain the greatest accuracy and then adjusted by eye using AliView^30^ to ensure sequences were in frame. Loci were then concatenated with *phyluce_align_concatenate_alignments* (Phyluce v 1.7)^31^.

### Phylogenetic analysis

Phylogenetic trees were constructed using the maximum likelihood method (ML) implemented within IQtree v2^32^ on 14 concatenated mt PCGs for Octocorallia and 13 mt PCGs for Scleractinia and Antipatharia, with ultrafast bootstrap approximation^33^ using 1000 replicates. The GTR + G substitution model was selected as best fit for the concatenated nucleotide sequences of Scleractinia mt PCGs based on the recent work by Seiblitz et al.^34^. The TVM + F + G4 and TVM + F + R5 substitution models were determined to be the best fit models for octocoral and antipatharian mt PCG concatenated sequences by ModelFinder^35^ within IQtree2. Phylogenetic trees were unrooted for use in the selection tests performed within Codeml^36^. Phylogenetic trees rooted for visualization can be found in the supplementary material (Supplementary Figs. S1, S2 and S3).

### Positive selection analysis

Adaptive evolution of each mt PCG of deep-sea and shallow-water octocorals, scleractinians, and antipatharians was separately evaluated using Codeml within the PAML package^36^ to estimate ω—the ratio of nonsynonymous (*dN*) to synonymous (*dS*) nucleotide substitutions. Three pairwise model comparisons were selected following Jeffares et al.^37^ and separately performed for each individual mt PCG region in each group of corals to assess various scenarios of positive selection. Inserted introns within *cox1* and *nad5* of Scleractinia and Antipatharia were removed and the flanking exonic regions were combined and evaluated as a single block.

The “branch” model comparisons (*one-ratio vs. two-ratio*) were used to detect whether deep-sea species, set as the foreground, were more likely to have a different ω from the shallow-water species (background), across branches or specific lineages in the phylogeny. This comparison included the *one-ratio* (M0) model, which estimated one average ω across the entire gene and served as the null against the *two-ratio* (M2) model which enabled ω to vary across the foreground and background branches^37^.

The “site” models (*nearly neutral vs. positive selection*) were compared to estimate positive selection and an ω ratio that was variable among codons (sites) but remained the same across lineages. These models differed from the others applied because detection of positive selection was not foreground-specific^37^. The *nearly neutral* (M1a) model, which served as the null, only allowed two classes of sites (0 < ω < 1, ω = 1) while the *positive selection* (M2a) model additionally allowed for positive selection (ω > 1).

Lastly, the “branch-site” models (*Model A null vs. Model A alternative*) were implemented to address more specifically whether mt genomes of deep-sea species were more likely to be under positive selection (ω > 1) than shallow-water corals. These models allow for an ω value that is variable across particular sites and branches. *Model A* (MA) *null* allowed only two classes of purifying or neutral selection (0 < ω < 1, ω = 1) for both the foreground and background species. This was compared to the MA *alternative* which allowed for positive selection (ω > 1) as a third class in the foreground species, while the background was restricted to two classes (ω = 0, ω = 1)^38^.

Significant differences between the pairwise model comparisons were determined by a likelihood ratio test (LRT) which identified whether the data better fit parameters of the null or alternative model in each comparison^37,39^. Adaptive evolution was inferred across each PCG in the “branch” models if the LRT was significant (p < 0.05) and ω was > 1. In the “branch-site” and “site” models adaptive evolution was inferred if the LRT was significant and sites of positive selection with a posterior probability > 95% were identified by the Bayes Empirical Bayes (BEB) output within those models^40^.

### Exploratory analyses

Additional analyses were performed to test potential factors influencing the detection of positive selection in mt genomes by modifying the foreground taxa or excluding specific taxa from the alignments before running the same models. Firstly, sea-pens within the superfamily Pennatuloidea^41^, occur at depths down to 6100 m. Among octocorals, this group includes some of the deepest-occurring species and has the second greatest number of species represented in the deep sea^42,43^. Therefore, other deep-sea octocorals (n = 11) were removed and deep-sea sea pens (n = 14) were exclusively selected as the foreground against shallow-water octocorals (n = 59) to assess whether the signal for adaptive evolution may arise at deeper depths. Secondly, the scleractinian mt alignments included three highly divergent species (*Letepsammia superstes, Letepsammia formosissima, and Rhombopsammia niphada*) belonging to the Micrabaciidae family. In phylogenies constructed from mt genes/genomes, these species are recovered as sister to all other scleractinians, and thus have been deemed the “basal clade”^34,44,45^, but this group is recovered as sister to complex corals in nuclear phylogenies^27,28^. To determine whether the incongruent placement of this family affected the outcome of these analyses, the three micrabaciids were excluded from the dataset and models rerun. Furthermore, Scleractinia is the only extant order of anthozoans that includes many colonial and solitary species, while Octocorallia and Antipatharia are primarily and strictly colonial^46^. To determine the influence of adaptive evolution on solitary vs. colonial corals in the deep sea, the foreground was modified to exclusively test solitary-deep (n = 8) scleractinians against shallow-water Scleractinia (n = 33) by removing colonial-deep representatives (n = 3) from the analysis. The reverse was accomplished by removing the solitary-deep representatives, while the colonial-deep scleractinians exclusively made up the foreground against shallow-water species. The list of sequences involved in these datasets can be found in Supplementary Table S1 online.

## Results

### Octocorallia

**Figure 1:**
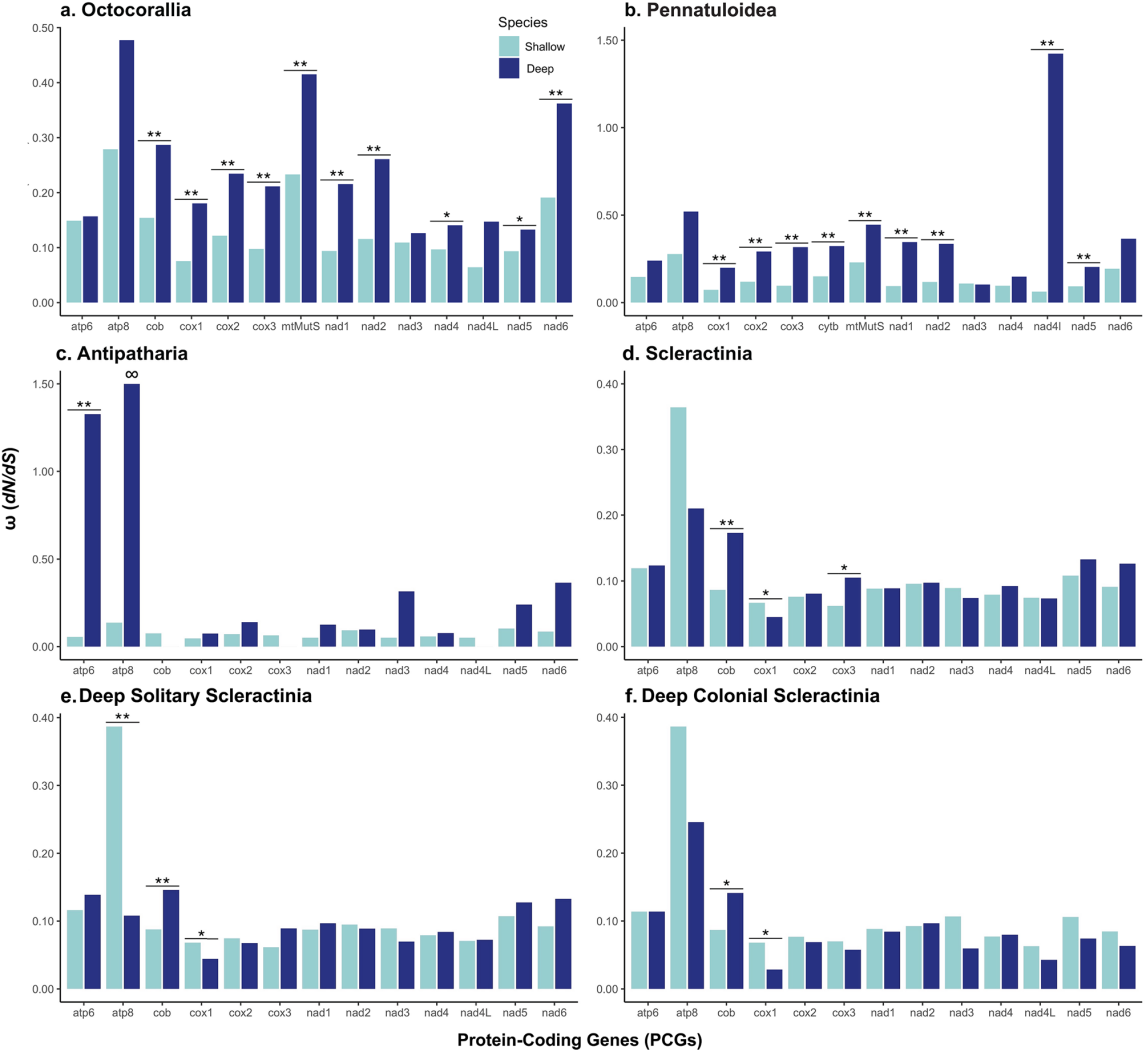
Average ω of each mt PCG estimated by branch-model analysis (M0 vs. M2) of deep- and shallow- water species of corals. Foreground set as deep-sea species of (**a**) Octocorallia, (**b**) Pennatuloidea, (**c**) Antipatharia, (**d**) Scleractinia, (**e**) Deep Solitary Scleractinia, (**f**) Deep Colonial Scleractinia *p < 0.05, **p < 0.01.

The branch-model analysis (M0 vs. M2), applied to determine whether deep-sea species were more likely to have a different ω ratio (*dN/dS*) than shallow-water species, revealed all PCGs in the mt genome of octocorals were under purifying selection (ω < 1). Average omega values of each gene ranged from 0.0697 to 0.2964 in the null, M0 model (Supplementary Table S2). However, pairwise comparison with the M2 model revealed that deep-sea species consistently had significantly (3.72E-15 < p < 0.0458, 3.99 < LRT < 61.84) higher ω ratios as compared to shallow-water species in the majority of the mt PCGs (Fig. 1a)*.* Among these genes, ω values ranged from 0.1264 to 0.4772 in deep-sea species and 0.0642 to 0.2790 in shallow-water species (Supplementary Table S2).

The site models (M1a vs. M2a) were applied to identify sites of positive selection (ω > 1) by assessing whether the data had a better fit to the M2a model, which allowed ω > 1. Sites of positive selection were inferred by the BEB analysis output of PCGs that were significantly different in the site models. Eight positively selected sites across *atp8* and *nad6* in octocorals were identified in the site-models (Table 1). The branch-site models (MA *null* vs. MA *alternative*) were performed to identify positive selection along lineages and codons particularly of deep-sea taxa. In deep-sea octocorals, 14 sites of positive selection with a posterior probability > 95% were identified across three genes *cox1, mtMutS,* and *nad1* (Table 2)*,* all of which were also significant in the branch-model (M0 vs. M2) analysis (Fig. 1a). *mtMutS* had the greatest number of sites under positive selection (Table 2).

**Table 1:** Sites under positive selection as determined by BEB analysis within the site models (M1a vs. M2a).

Foreground	Gene	2ΔlnL	p-value	Codon	Amino acid^a^	BEB
Octocorallia (n = 25)	*atp8*	17.21	0.0002	19	T	0.989*
				51	S	0.968*
				60	S	0.990*
	*nad6*	121.80	3.56E−27	204	–	1.000**
				205	–	1.000**
				206	–	1.000**
				207	–	0.999**
				208	–	1.000**
Pennatuloidea (n = 14)	*nad6*	69.18	9.51E−16	204	–	1.000**
				205	–	1.000**
				206	–	0.995**
				207	–	0.969*
				208	–	0.969*

Amino acid refers to IUPAC amino acid codes.

*BEB > 95%, **> 99%.

^a^Amino acids listed are in reference to the first sequence in alignment; “–” denotes a gap at that codon.

**Table 2:** Sites under positive selection in deep-sea corals as determined by BEB analysis within branch-site models (MA *null* vs. MA *alternative*).

Foreground	Gene	2ΔlnL	p-value	Codon	Amino acid^a^	BEB
Octocorallia (n = 25)	*cox1*	5.63	0.0177	458	C	0.988*
				522	L	0.990**
	*mtMutS*	69.37	8.16E−17	84	G	0.988*
				546	H	0.982*
				547	L	0.998**
				551	H	0.999**
				655	M	0.965*
				656	P	1.000**
				657	–	0.988*
				1037	–	0.993**
				1105	C	0.958*
				1111	–	1.000**
	*nad1*	21.20	4.13E−06	118	I	1.000**
				126	I	1.000**
Pennatuloidea (n = 14)	*atp8*	4.23	0.0398	60	S	0.960*
	*cox1*	8.53	0.0035	28	S	0.980*
				208	I	0.987*
				522	L	0.989*
				523	S	0.950*
	*cob*	7.23	0.0071	33	Y	0.998**
	*mtMutS*	111.85	3.86E−26	61	S	0.965*
				536	L	0.994**
				540	H	0.999**
				541	L	0.975*
				643	V	0.998**
				644	M	1.000**
				645	P	1.000**
				646	–	1.000**
				1036	S	0.989*
	*nad1*	21.49	3.55E−06	118	I	0.991**
				126	I	1.000**
				323	A	0.995**
	*nad2*	11.49	0.0001	93	Q	0.994**
				282	M	1.000**
				384	S	0.987*
	*nad4l*	3.91	0.0481	92	L	0.951*
Antipatharia (n = 6)	*nad3*	5.21	0.0225	96	V	0.954*

Amino acid refers to IUPAC amino acid codes.

*BEB > 95%, **> 99%.

^a^Amino acids listed are in reference to first sequence in alignment; “–” denotes a gap at that codon.

Influence of positive selection at greater depths was also evaluated by restricting the foreground species to deep-sea sea pens (excluding other deep-sea octocorals) and the background to shallow-water octocorals. The branch models found that all PCGs that were significant in the full Octocorallia analysis, remained significantly different (6.16E-10 < p < 0.0007; 10.20 < LRT < 38.27) between deep-sea sea pens and shallow octocorals with the same trends except for *nad4* and *nad6,* which were no longer significant based on the LRT (Fig. 1b, Supplementary Table S3). In addition, the LRT detected *nad4l* to be significant (p = 0.0014; LRT = 10.20) when excluding other deep-sea octocorals, with a ω ratio > 1 in deep-sea sea pens (ω = 1.4229; Fig. 1b, Supplementary Table S3). The site models indicated *nad6* was better fit to M2a and identified five sites of positive selection within the PCG (Table 1). Lastly, the branch-site analysis detected 22 possible sites of positive selection with a posterior probability > 95% across seven genes in Pennatuloidea: *atp8, cox1, cob, mtMutS, nad1-2, and nad4l. mtMutS* again had the greatest number of possible sites for positive selection (Table 2).

### Antipatharia

Omega values for antipatharians ranged from 0.0474 to 0.1460 in the M0 model of the branch-model analysis (Supplementary Table S2). *atp6* was the only gene to be significantly different (p = 0.0034; LRT = 8.57) between deep-sea and shallow-water antipatharians (Fig. 1c). Additionally, *atp6* displayed an ω ratio > 1 (ω = 1.3263) in deep-sea species against shallow-water species (ω = 0.0560) undergoing purifying selection. Omega values ranged from 0.0001 to 999 (otherwise infinity) in deep-sea species, with the extreme high value (999) resulting from no synonymous substitutions in *atp8* and the extreme low value (0.0001) resulting from no nonsynonymous substitutions in *cob, cox3,* and *nad4l* (LRT did not determine significance among these genes. Omega values ranged 0.0469 to 0.1371 in shallow-water species (Supplementary Table S2, Fig. 1c). There were no significant differences found in the site models for antipatharians. However, the branch-site analysis, which allowed an ω value that is variable across branches and sites, detected one site of positive selection within the *nad3* gene of deep-sea taxa (Table 2).

### Scleractinia

All scleractinian mt PCGs were under purifying selection (ω < 1) in the branch-model analysis. Omega values ranged from 0.0620 to 0.3123 in the M0 model (Supplementary Table S2). Deep-sea species were significantly more likely to exhibit ω ratios that differed from the background in *cob* (p = 1.51E-5, LRT = 18.72) and *cox3* (p = 0.0128, LRT = 6.20; Fig. 1d, Supplementary Table S2)*.* Omega values ranged from 0.0451 to 0.2101 in deep-sea species and 0.0622 to 0.3645 in shallow-water species (Supplementary Table S2). *Cox1* was also significant (p = 0.0136, LRT = 6.09) but displayed the opposite relationship, with shallow-water species having significantly higher ω values than deep-sea species (Fig. 1d, Supplementary Table S2). There were no significant differences between deep-sea and shallow-water species from the branch-site (MA *null* vs MA *alternative*) and site-model (M1a vs M2a) analyses signifying that positive selection was undetected even at the codon level. Excluding the highly divergent, basal species (*L. superstes, L. formosissima,* and *R. niphada*) did not affect the outcome of these analyses (Supplementary Fig. S4).

Additional analyses applied to assess the influence of positive selection on colonial versus solitary species in Scleractinia revealed that in both cases, *cob* (0.0052 < p < 0.0272, 4.88 < LRT < 7.79) and *cox1* (3.98E-5 < p < 0.0103, 6.57 < LRT < 16.88) remained significantly likely to display different ω ratios (the same trend) among deep-sea and shallow-water species (Fig. 1e,f, Supplementary Table S3). In addition, *atp8* had a significantly (p = 0.0022, LRT = 9.36) higher ω ratio (0.3870) in shallow-water species when deep, solitary Scleractinia were set as the foreground species (Supplementary Table S3, Fig. 1e). Again, there were no significant differences in the branch-site and site models in these additional analyses for Scleractinia.

## Discussion

The geographical expanse of corals from coastal reefs to the abyssal depths of the deep sea allude to their molecular diversity. This is especially true of their mitochondrial genomes which encode for metabolic functions supportive of their success under extreme conditions^47^. There have been numerous studies assessing positive selection within the mt genome of various Metazoa along environmental gradients^48–51^ and of deep-sea invertebrates specifically^4,13,14,52–54^. However, the uniquely low variability and slow evolution of coral (Anthozoa) mtDNA^15,16^ might create a discontinuity between what we know of selection pressures in other metazoan mt genomes versus its influence in corals. By using phylogenies constructed from mt genomes generally congruent with previously published mt-based phylogenies^20,25,34^, we address this knowledge gap and broaden our understanding of adaptation in the deep sea by providing insight into mitochondrial evolution as a function of depth in three diverse coral groups.

### Patterns of purifying selection

Branch-model analyses indicated that all mt PCGs of deep-sea and shallow-water species within Octocorallia, Scleractinia, and Antipatharia (with the exception of *atp6*) underwent strong purifying selection. The low ω (*dN/dS*) values along branches in the phylogeny indicated a greater average number of synonymous substitutions revealing that overall evolution favored mutations that did not alter the encoded amino acids. Given the functional metabolic importance of the mt genome in OXPHOS, this strong purifying selection likely acts to prevent the fixation of deleterious mutations^55^. Additionally, among octocorals deep-sea species displayed higher ω values in all mt PCGs and therefore underwent more relaxed purifying selection in comparison to their shallow-water counterparts.

Of the 14 mt PCGs in octocorals, 10 were better fit to the M2 model that allowed for positive selection, indicating that this difference in ω values in deep-sea corals is not random. A similar trend of overall purifying selection, but higher nonsynonymous mutations in deep-sea lineages, has been observed in other deep-sea invertebrates such as bathymodioline mussels, vesicomyid clams, and alvinocaridid shrimp, all of which had representatives from hydrothermal vent environments included in their studies^13,14,56^. In a similar manner, Tibetan loaches and galliform birds subjected to the extreme selective pressures of a high-altitude environment also displayed higher average ω ratios (but < 1) than in non-Tibetan species^49,50^. The observation of purifying selection, yet higher numbers of nonsynonymous mutations, in deep-sea octocorals provides additional evidence for mitochondrial adaptation towards a relaxed purifying selection, even amongst the slow evolution of anthozoan mt genomes that sets them apart from other Metazoa^15^. Perhaps these nonsynonymous substitutions give selective advantages in deep-sea lineages under extreme environmental conditions.

In contrast to octocorals, branch-model analysis for Scleractinia and Antipatharia indicated that only three (*cob, cox1,* and *cox3*) and one (*atp6*) of the 13 mt PCGs were significantly different between deep and shallow-water species. *cob* and *cox3* had significantly higher ω values in deep-sea lineages of Scleractinia which is consistent with the trend in octocoral PCGs. However, in Scleractinia, *cox1* displayed a greater ω value in the shallow-water species, opposing the previous observations in octocorals. Similarly, *cox1* also had a higher ω ratio in shallow-water mussels—the only exception to the trend previously described in the bathymodioline mussel mt genome^14^. *cox1*, a subunit of cytochrome c oxidase complex IV, functions in facilitating an electron transfer to oxygen as the ultimate acceptor molecule in the generation of ATP through oxidative phosphorylation^12,47^. Repetition of this finding in these two studies highlights that *cox1* may potentially be under relaxed purifying selection in shallow water lineages because of other stressors not as common in the deep sea, such as oxidative stress, which has been noted to impact shallow scleractinians^57^. Nevertheless, future studies could explore selection pressures on *cox1* in shallow-water scleractinians.

### Signatures of positive selection

The *atp6* gene in deep-sea Antipatharia was an exception to the overall purifying selection observed in the branch-models, as it was the only group to display ω > 1 suggesting selection pressures are at play to meet the metabolic demands of the deep-sea environment. The *atp6* gene has a vital role in OXPHOS through encoding the F_0_ rotator region of ATP synthase (complex V), which transfers the energy created by the proton electrochemical gradient to the F_1_ region to phosphorylate ADP to ATP^58^. *atp6* was found to have sites of positive selection in studies previously mentioned for the deep-sea anemone *Bolocera* sp*.,* bathymodioline mussels, and alvinocaridid shrimp^4,13,14^. However, our finding is unique in that it shows deep-sea black coral species are more likely to be under positive selection across *atp6* in contrast to only at a few select codon sites. This result encourages further exploration of the evolutionary differences in the *atp6* gene in black corals as they were the only group to show positive selection within the branch-models. Notably, black corals are some of the longest living and deepest occurring corals in the deep sea^9,59^.

Although purifying selection was depicted as the predominant driver of mitochondrial evolution in the branch-models of octocorals, the site-models indicated that positive selection occurred at eight sites within *atp8* and *nad6*. We also found evidence of positive selection more specifically in deep-sea taxa of Octocorallia at 14 sites across *cox1, mtMutS,* and *nad1,* and at one site in *nad3* of deep-sea Antipatharia. Thus, we present the first evidence for adaptive evolution of octocoral and black coral mt genes in the deep sea through selection analyses. These positively selected sites may influence physiological changes in the bioenergetic functions they encode. *nad1, nad3,* and *nad6* are three of the seven mt-encoded subunits that comprise four membrane-bound proton pumps within the NADH dehydrogenase enzyme (complex I), the first and largest complex in the respiratory chain^60^. Positively selected sites along the NADH dehydrogenase genes have been detected in all of the mt selection studies of deep-sea invertebrates previously mentioned and in a deep-sea crab, holothurian, and asteroid mt genome^4,13,14,52–54^. Combined with our results, there is strong evidence that these genes have important metabolic functions for deep-sea fauna because of continued detection of positively selected sites across diverse taxa. Moreover, positively selected sites in *cox1* may be advantageous in deep-sea lineages to support ATP production by OXPHOS to meet high metabolic demands of the deep sea^61^. The *atp8* gene*,* the second PCG for ATP synthase, further supports the assembly of the F_0_ region of the enzyme and therefore facilitates ATP production^58,61^ Because of its function, substitutions in genes encoding for complex V may have significant impacts on the metabolic capacities of an organism to adapt to the bioenergetic demands of extreme environments.

Lastly, the unique presence and functionality of *mtMutS* in mt genomes of octocorals are of growing interest among researchers^18,62,63^. Most recently, using the same data sequences applied here (from Quattrini et al.^27^) to capture mt genomes, Muthye et al.^19^ demonstrated a putative loss of *mtMutS* in *Pseudoanthomastus* sp. 1 and a higher rate of mt-sequence evolution compared to closely related species with *mtMutS*. This supports the ongoing case for the function of *mtMutS* in DNA repair^18,62,63^. The results of our study further demonstrate that the *mtMutS* gene is significantly more likely to contain sites of positive selection in deep-sea lineages of octocorals based on a significant LRT of the branch-site model. Perhaps the metabolic requirements of the deep sea demand greater DNA repair capabilities and/or functional requirements of *mtMutS*.

### Further considerations for selection analysis

Incongruence was evident between the results of the branch-site and site models applied in this study. However, this can be attributed to episodic selection, meaning positive selection may only be acting on specific sites and lineages which could be overshadowed in the branch-models by the overall purifying selection occurring across the genome^37,64,65^. This would support why genes with positively selected sites also displayed strong purifying selection in the branch-models. However, the *atp6* gene of Antipatharia was significantly more likely to have an average ω > 1 across deep-sea lineages based on the branch-models. *atp6* was therefore expected to have sites of positive selection in the branch-site models since the gene overall had an average ω under positive selection. The BEB analysis identified one site of positive selection (130, M, 0.951*) in deep-sea Antipatharia with a posterior probability > 95%, yet the LRT was not significant. A statistical study on accurately detecting sites of positive selection revealed that when 10% of sites are under strong positive selection, a 30-taxon tree input within Codeml correctly identifies over 75% of positively selected sites while a 5-taxon tree only detects about 20% of positively selected sites^66^. This suggests that our smaller sample size for black corals (n = 13) could be limiting the detection of positively selected sites in the branch-site model. However, there are only an estimated 273 species within the order Antipatharia^25^ in comparison to the over 3500 and 1500 species within Octocorallia and Scleractinia^41,67^. Although our black coral tree included the fewest taxa, it had the highest sampled proportion of species (4.76%) among these groups and nonetheless conveyed the bathymetric expanse of the order by including the shallowest and deepest species for which the mt genomes were available. The impressive depth distribution of Antipatharia and limited knowledge of this group encourages further analysis of their adaptive evolution as more evidence may be uncovered with the inclusion of more black coral mt genomes.

In this study, deep-sea corals were generalized as species inhabiting > 200 m depth, yet we know that corals can be found down to abyssal depths^8,9,68^. It is possible that signals for positive selection may arise at deeper depths and the inclusion of the upper bathyal fauna may obscure their detection. We selected a subset of octocorals that included 14 deep-sea sea-pens, generally regarded as deeper dwelling species within the group^43^, to test for variability in the results. Results revealed that *nad4l* had an average ω > 1 in deep-sea sea-pens, which was not detected when other deep-sea octocorals were included in the foreground. Additionally, there were eight more sites of positive selection detected within Pennatuloidea in branch-site analysis, but three fewer in the site-model analysis. This presents a future avenue for positive selection analyses to be conducted across various depth ranges to map changes in adaptive evolution associated with environmental extremes, such as in the abyss.

## Conclusion

The results of this study strongly support that the evolution of mt PCGs of deep and shallow- water species of Octocorallia, Scleractinia, and Antipatharia is mainly driven by purifying selection, suggesting that mitochondrial function within corals is highly conserved. However, *atp6* in black corals and *nad4l* in sea pens were exceptions to this trend with average ω values indicative of positive selection. Sites of positive selection were located at 14 codon sites in *cox1, mtMutS,* and *nad1* of deep-sea octocorals and at one site in *nad3* of deep-sea antipatharians supporting the influence of selective pressures in the deep-sea environment acting on these genes. Lastly, analyses presented within this study emphasize the variability among adaptive evolution in the mt genome in different taxonomic groups of corals. The ability of corals to inhabit such an extensive range of depths is in large part due to the metabolic functions encoded by the mitochondrial genome. This study provides further insight into specific mitochondrial PCGs under adaptive evolution in Octocorallia, Scleractinia, and Antipatharia inhabiting the deep sea.

## Supplementary Information

Supplementary Information.

Supplementary Table 1.

## Data Availability

Mitochondrial gene alignments are available on GenBank; numbers can be found in Supplemental Table 1. Code for analyses, trees, and alignments are on GitHub: https://github.com/ninramos/deepsea-coral-mitogenome-selection.
