# Supplementary material for: Selection in coral mitogenomes, with insights into adaptations in the deep sea
Source: Sci Rep. 2023 Apr 12;13:6016. doi: 10.1038/s41598-023-31243-1 (PMC10097804; doi:10.1038/s41598-023-31243-1)
Supplement: Supplementary file 1 — Supplementary Information. [file 41598_2023_31243_MOESM1_ESM.pdf]

Supplementary Information

Selection in coral mitogenomes, with insights into adaptations in the deep sea

Nina I. Ramos[1], Danielle M. DeLeo[1], Jeremy Horowitz[1], Catherine S. McFadden[2], Andrea M. Quattrini[1,*]

[1] Department of Invertebrate Zoology, National Museum of Natural History, Smithsonian Institution, Washington, DC, 20560, USA
[2] Department of Biology, Harvey Mudd College, Claremont, CA, 91711, USA

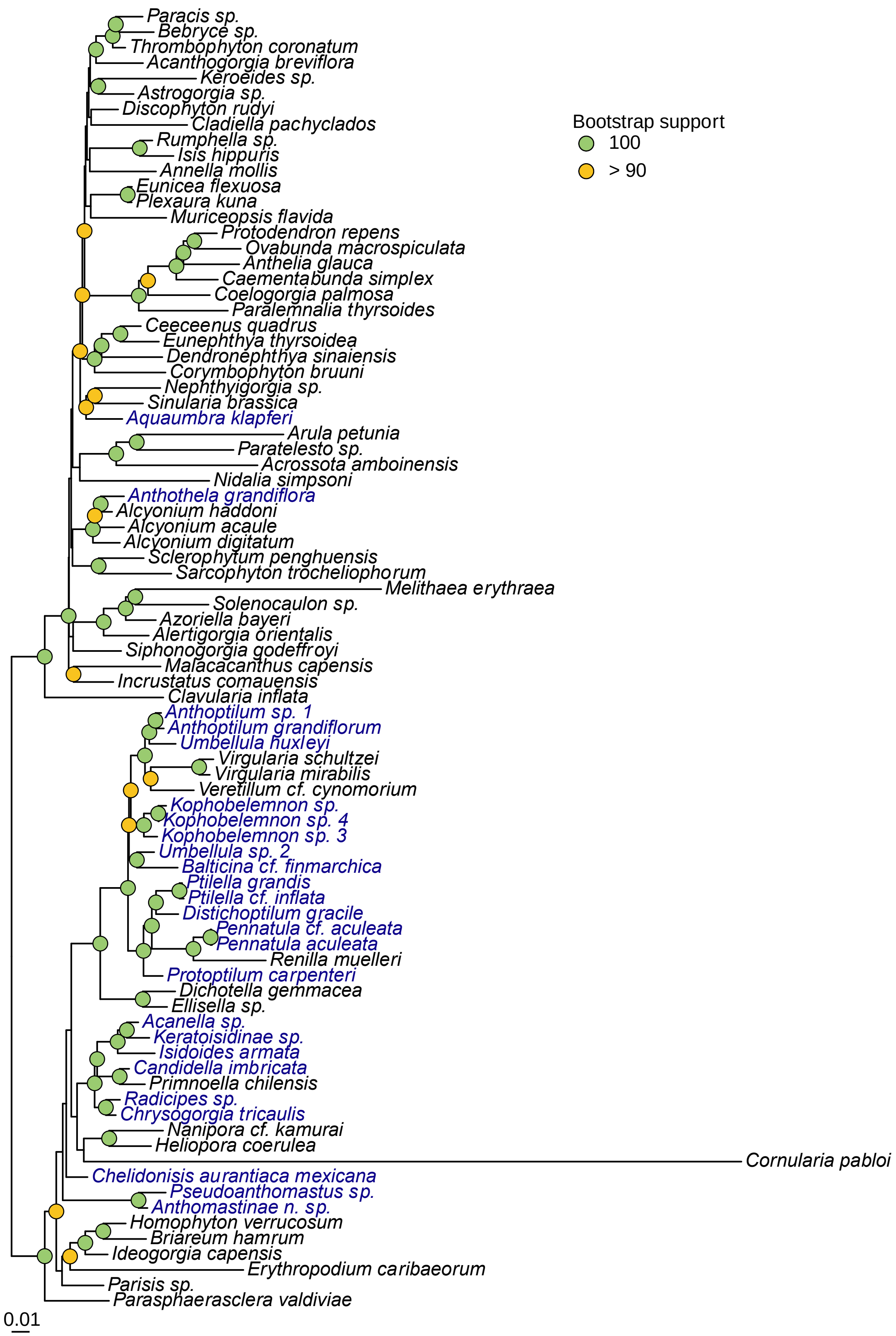

Supplementary Figure S1. Maximum likelihood tree of Octocorallia (total n=84) based on 14 mt PCGs; deep-sea octocorals (blue, n=25). Phylogenetic tree was rooted for visualization to the Scleralcyonacea clade as prior studies (Quattrini et al. 2020, McFadden et al. in press) consistently recovered this clade as reciprocally monophyletic with the Malacalcyonacea. Bootstrap support values are displayed for each node.

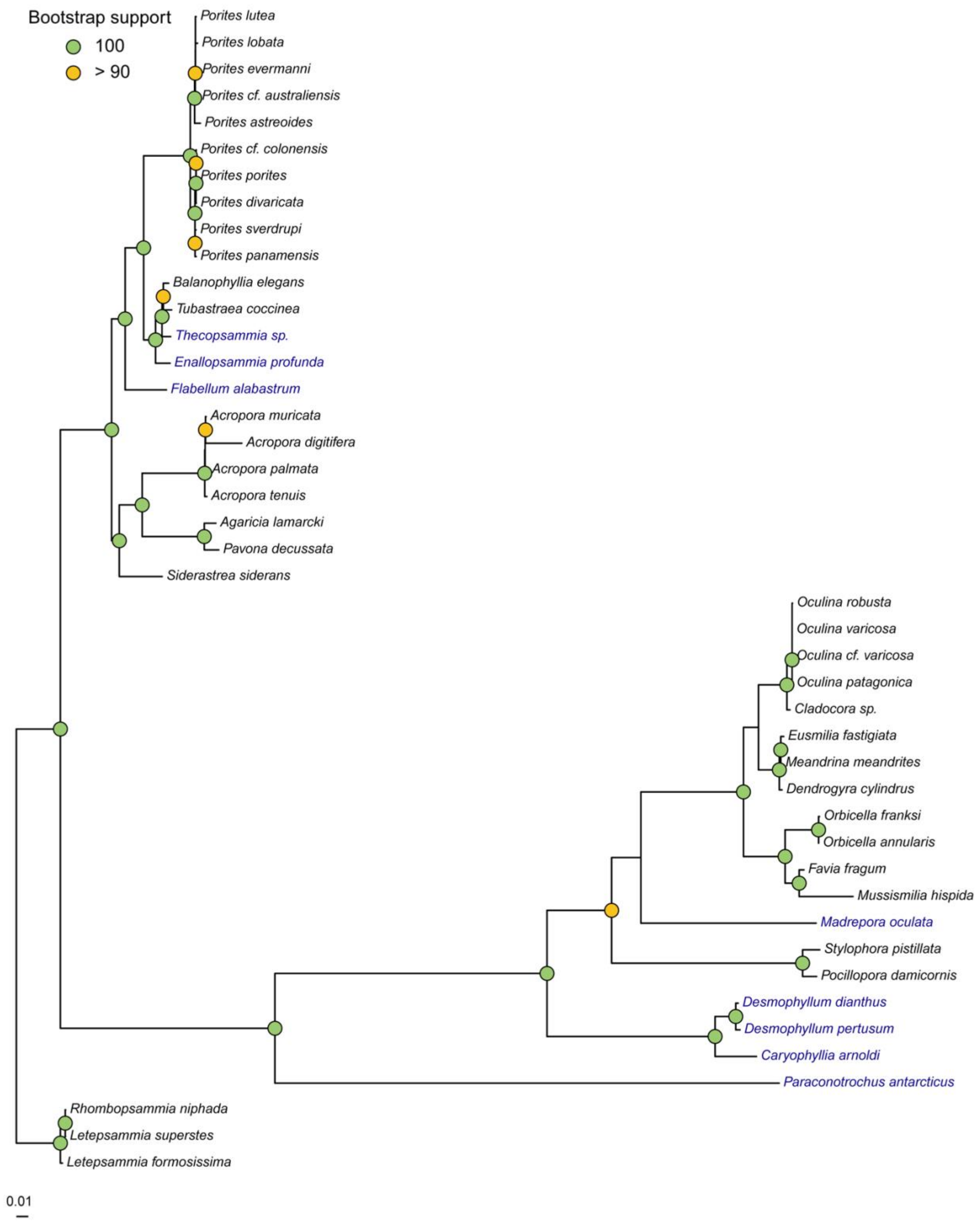

Supplementary Figure S2. Maximum likelihood tree of Scleractinia (total n=44) based on 13 mt PCGs, deep-sea Scleractinia (blue, n=11). Tree is rooted to the basal clade for visualization following Kitahara et al. (2010), Campoy et al. (2020), and Seiblitz et al. (2020). Bootstrap support values are displayed for each node.

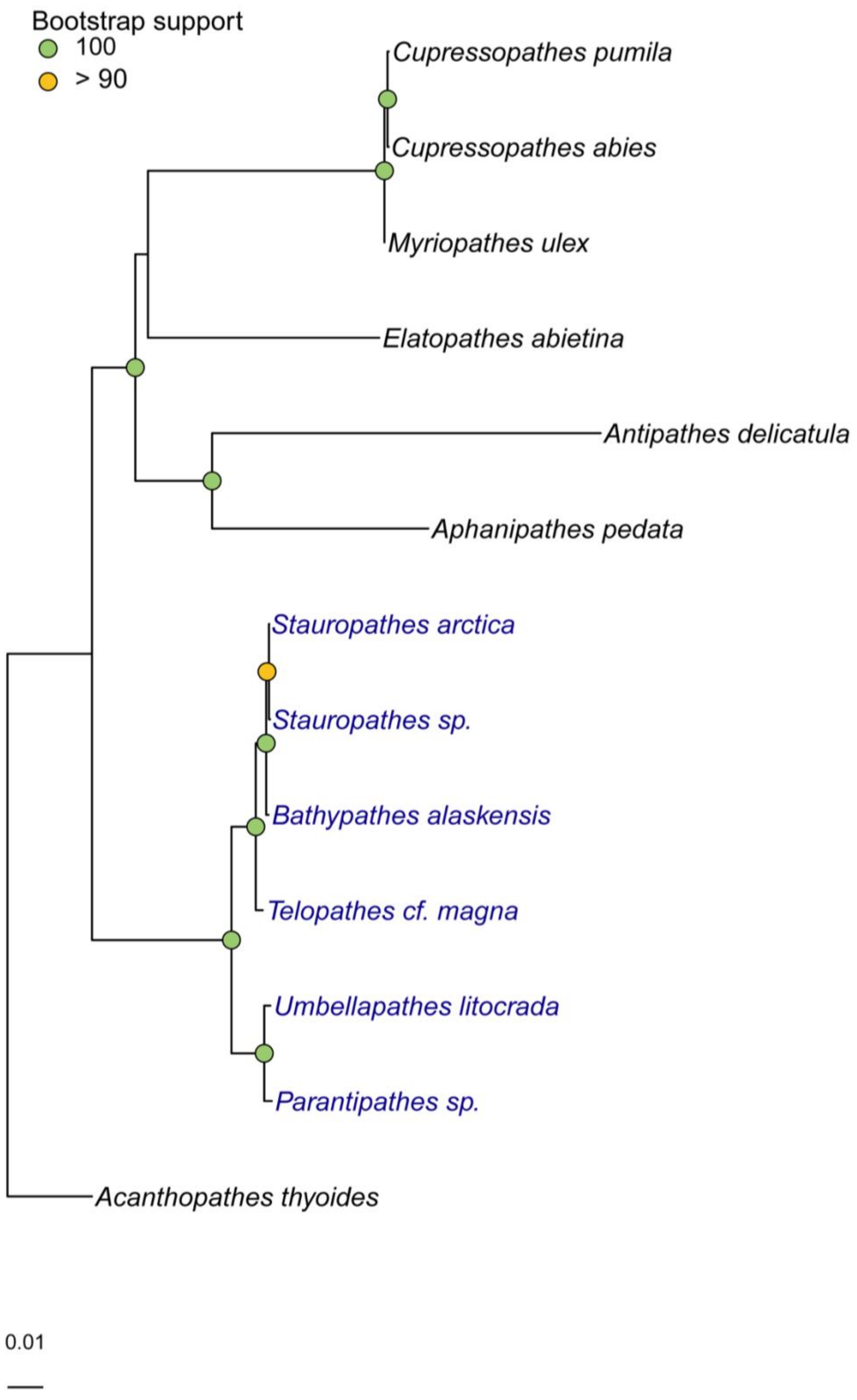

Supplementary Figure S3. Maximum likelihood tree of Antipatharia (total n=13) based on 13 mt PCGs, deep-sea antipatharians (blue, n=6). Tree is rooted at the midpoint for visualization and bootstrap support values are displayed for each node.

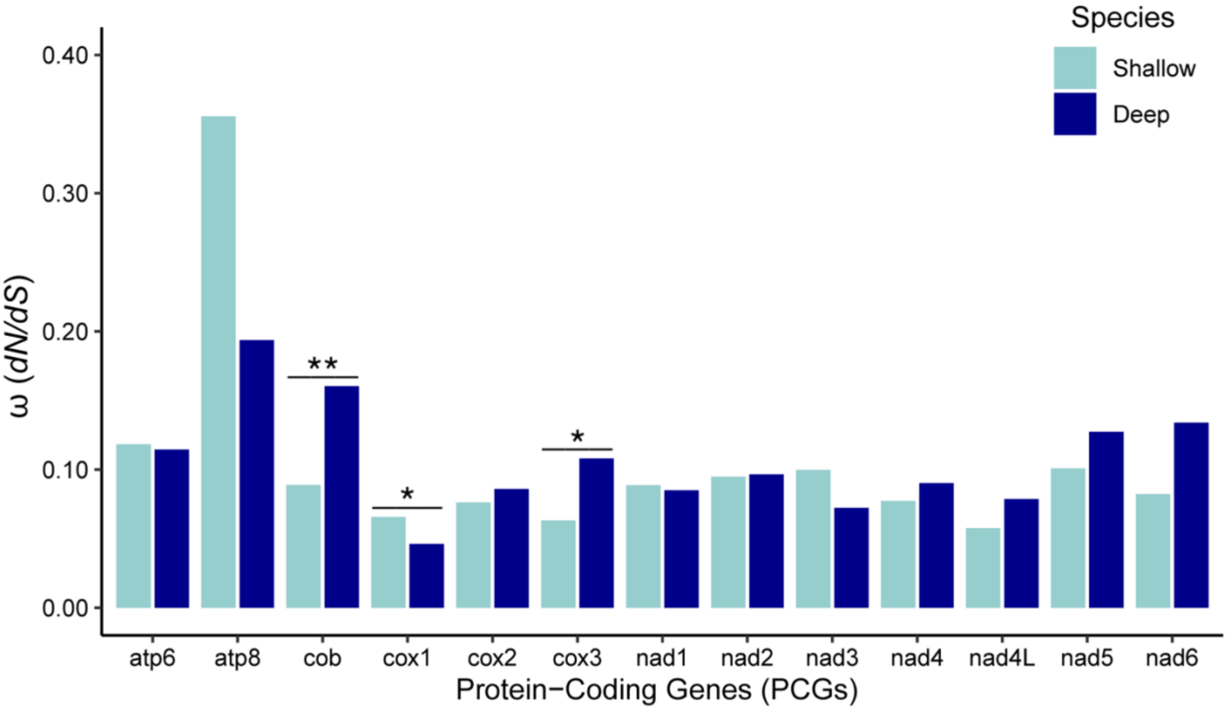

Supplementary Figure S4. Estimated average ω in each mt PCG calculated in branch-model analysis of deep- (n=8) and shallow- water species (n=33) of Scleractinia (total n=41) with basal species removed (n=3).

Supplementary Table S2. Estimated ω values from the branch-model analysis (M0 vs M2) for deep-sea and shallow-water Octocorallia, Scleractinia, and Antipatharia.

Foreground	Gene	2ΔlnL	p-value	ω (M0)	$\omega_{shallow}$ (M2)	ω_{deep} (M2)
Octocorallia (n=25)	*atp6*	0.04	0.8473	0.1492	0.1489	0.1567
	atp8	3.05	0.0807	0.2965	0.2790	0.4772
	cob	23.20	1.46E-06	0.1637	0.1541	0.2868
	cox1	33.90	5.81E-09	0.0824	0.0754	0.1804
	cox2	11.11	0.0009	0.1303	0.1216	0.2343
	cox3	15.79	0.0001	0.1073	0.0978	0.2113
	mtMutS	61.84	3.72E-15	0.2451	0.2330	0.4151
	nad1	15.54	0.0001	0.1012	0.0941	0.2155
	nad2	18.38	1.81E-05	0.1222	0.1156	0.2608
	nad3	0.45	0.5045	0.1104	0.1091	0.1264
	nad4	3.99	0.0458	0.0992	0.0969	0.1405
	nad4l	3.73	0.0535	0.0698	0.0642	0.1472
	nad5	5.30	0.0214	0.0960	0.0936	0.1327
	nad6	12.71	0.0004	0.2089	0.1910	0.3622
Scleractinia (n=11)	*atp6*	0.03	0.8619	0.1205	0.1196	0.1237
	atp8	2.45	0.1173	0.3123	0.3645	0.2101
	cob	18.72	1.51E-05	0.1017	0.0863	0.1730
	cox1	6.09	0.0136	0.0620	0.0666	0.0451
	cox2	0.05	0.8209	0.0770	0.0762	0.0805
	cox3	6.20	0.0128	0.0700	0.0622	0.1051
	nad1	0.00	0.9769	0.0884	0.0883	0.0888
	nad2	0.01	0.9267	0.0961	0.0958	0.0974
	nad3	0.24	0.6221	0.0864	0.0893	0.0743
	nad4	0.98	0.3224	0.0816	0.0792	0.0925
	nad4l	0.00	0.9639	0.0742	0.0745	0.0733
	nad5	2.04	0.1534	0.1127	0.1083	0.1329
	nad6	-3.15*		0.0975	0.0910	0.1265
Antipatharia (n=6)	*atp6*	8.57	0.0034	0.0617	0.0560	1.3263
	atp8	1.06	0.3031	0.1460	0.1371	999.0000
	cob	2.79	0.0948	0.0741	0.0757	0.0001
	cox1	0.33	0.5684	0.0474	0.0469	0.0752
	cox2	0.55	0.4596	0.0733	0.0719	0.1399
	cox3	1.22	0.2701	0.0627	0.0637	0.0001
	nad1	0.50	0.4795	0.0523	0.0515	0.1259
	nad2	0.00	0.9498	0.0935	0.0934	0.0983
	nad3	1.44	0.2301	0.0534	0.0507	0.3160
	nad4	0.11	0.7374	0.0595	0.0591	0.0775
	nad4l	0.65	0.4212	0.0500	0.0515	0.0001
	nad5	1.57	0.2097	0.1050	0.1032	0.2396
	nad6	0.92	0.3362	0.0884	0.0868	0.3650

*a negative LRT statistic reflects a potential numerical issue within the ML iteration within the Codeml program, however multiple runs in this case showed stable results.

Supplementary Table S3. Estimated omega ω values from the branch-model exploratory analysis (M0 vs M2) of deep-sea and shallow-water Pennatuloidea, Scleractinia sans basal species, solitary, and colonial Scleractinia.

Foreground	Gene	2ΔlnL	p-value	ω (M0)	$\omega_{shallow}$ (M2)	ω_{deep} (M2)
Pennatuloidea (n=14)	*atp6*	1.22	0.2687	0.1487	0.1473	0.2400
	atp8	2.33	0.1272	0.2895	0.2775	0.5209
	cob	19.44	1.04E-5	0.0768	0.0733	0.1990
	cox1	11.40	0.0007	0.1255	0.1190	0.2926
	cox2	19.76	8.80E-6	0.1038	0.0961	0.3178
	cox3	19.16	1.20E-5	0.1569	0.1505	0.3235
	mtMutS	38.27	6.16E-10	0.2371	0.2305	0.4452
	nad1	19.08	1.26E-5	0.1003	0.0946	0.3455
	nad2	13.81	0.0002	0.1219	0.1179	0.3356
	nad3	0.01	0.9360	0.1093	0.1094	0.1039
	nad4	2.41	0.1205	0.0978	0.0966	0.1485
	nad4l	10.20	0.0014	0.0668	0.0626	1.4229
	nad5	13.02	0.0003	0.0957	0.0931	0.2043
	nad6	3.10	0.0782	0.1975	0.1931	0.3653
Scleractinia sans basal (n=8)	*atp6*	0.03	0.8669	0.1174	0.1184	0.1145
	atp8	2.66	0.1030	0.2947	0.3556	0.1937
	cob	13.73	0.0002	0.1034	0.0889	0.1604
	cox1	4.84	0.0278	0.0612	0.0657	0.0462
	cox2	0.23	0.6344	0.0783	0.0763	0.0859
	cox3	6.37	0.0116	0.0717	0.0632	0.1080
	nad1	0.04	0.8326	0.0878	0.0887	0.0851
	nad2	0.01	0.9204	0.0953	0.0949	0.0967
	nad3	0.68	0.4098	0.0933	0.0997	0.0723
	nad4	0.95	0.3310	0.0797	0.0772	0.0902
	nad4l	0.54	0.4628	0.0627	0.0576	0.0787
	nad5	2.39	0.1220	0.1059	0.1009	0.1273
	nad6	-1.51*		0.0917	0.0823	0.1339
Deep solitary Scleractinia (n=8)	*atp6*	0.75	0.3850	0.1206	0.1163	0.1390
	atp8	9.36	0.0022	0.3004	0.3870	0.1080
	cob	7.79	0.0052	0.0964	0.0879	0.1459
	cox1	6.57	0.0103	0.0638	0.0684	0.0442
	cox2	0.09	0.7679	0.0738	0.0746	0.0676
	cox3	1.78	0.1820	0.0647	0.0615	0.0894
	nad1	0.25	0.6183	0.0891	0.0874	0.0969
	nad2	0.10	0.7572	0.0939	0.0949	0.0891
	nad3	0.26	0.6104	0.0867	0.0893	0.0699
	nad4	0.12	0.7304	0.0799	0.0791	0.0841
	nad4l	2.42E-3	0.9608	0.0711	0.0708	0.0723
	nad5	1.11	0.2926	0.1101	0.1073	0.1274
	nad6	-2.55*		0.1000	0.0924	0.1329
Deep colonial Scleractinia (n=3)	*atp6*	0.00	1.000	0.1138	0.1138	0.1138
	atp8	0.61	0.4359	0.3623	0.3865	0.2458
	cob	4.88	0.0272	0.0941	0.0869	0.1411
	cox1	16.88	3.98E-5	0.0606	0.0683	0.0287

cox2	0.13	0.7216	0.0752	0.0768	0.0688
cox3	0.60	0.4376	0.0670	0.0700	0.0579
nad1	0.03	0.8601	0.0876	0.0883	0.0844
nad2	0.03	0.8522	0.0934	0.0927	0.0967
nad3	1.73	0.1884	0.0969	0.1067	0.0595
nad4	0.03	0.8619	0.0776	0.0772	0.0799
nad4l	0.44	0.5086	0.0580	0.0630	0.0426
nad5	3.39	0.0656	0.0996	0.1061	0.0744
nad6	-4.10*		0.0811	0.0847	0.0633

*a negative LRT statistic reflects a potential numerical issue within the ML iteration within the Codeml program, however multiple runs in this case showed stable results.
